# Beyond AUC: a clinician’s guide to building and trusting prediction models in oncology—a narrative review

**DOI:** 10.3389/fonc.2026.1798803

**Published:** 2026-07-09

**Authors:** Xuexing Wang, Youxian Dou, Yufeng Wang, Kai Sun, Guozhong Zhou

**Affiliations:** 1 Department of Oncology, Anning First People’s Hospital Affiliated to Kunming University of Science and Technology, Anning, China; 2 Department of Oncology, Ganzhou Cancer Hospital, Ganzhou, Jiangxi, China; 3 Faculty of Medicine, Kunming University of Science and Technology, Kunming, China; 4 Cadre Medical Department, Third Affiliated Hospital of Kunming Medical University, Kunming, China; 5 Pain Management Department, Anning First People’s Hospital Affiliated to Kunming University of Science and Technology, Anning, China

**Keywords:** calibration, decision curve analysis, external validation, machine learning, neoplasms, nomograms, precision medicine

## Abstract

**Background:**

Prediction models are central to advancing precision oncology, yet many fail to translate into clinical practice due to methodological flaws and inadequate validation. This review provides a practical, clinician-oriented guide to the statistical principles and advanced methods for developing, validating, and interpreting robust prediction models.

**Methods:**

This narrative review used a targeted literature search of PubMed, Embase, and Web of Science to identify methodological papers, reporting guidelines, and representative oncology prediction model studies, with a focus on literature published between January 1, 2005, and February 28, 2025. Landmark methodological papers published before 2005 were also included when directly relevant. Rather than performing a systematic review or meta-analysis, we synthesized key statistical principles and illustrative examples to guide clinicians and researchers through model development, validation, interpretation, and clinical translation.

**Findings:**

A multifaceted evaluation encompassing discrimination, calibration, clinical utility, and external validation is essential for prediction models. Over-reliance on discrimination metrics such as the area under the receiver operating characteristic curve (AUC), while neglecting calibration and clinical utility, can lead to misleading conclusions about a model’s value. Rigorous external validation in geographically or temporally distinct cohorts is the most direct test of generalizability, and performance degradation should be interpreted through root-cause analysis rather than treated simply as model failure. Key challenges include managing overfitting, selecting appropriate modeling and validation strategies for different oncology scenarios, addressing special settings such as rare tumors and real-world data, and improving the interpretability of complex “black-box” models.

**Conclusion:**

Building a trustworthy prediction model requires a combination of advanced computational methods and rigorous statistical principles. To bridge the gap from model development to clinical impact, researchers must prioritize comprehensive validation, transparent reporting, scenario-appropriate modeling decisions, and critical assessment of a model’s real-world utility.

## 1 Introduction

In the era of precision medicine, particularly in oncology, prediction models have become a critical bridge between basic research and clinical practice (1). These models leverage patient data—spanning clinical, imaging, pathological, and multi-omics sources—to enable individualized predictions for disease risk, early diagnosis, prognostic stratification, and treatment response (2, 3). For instance, risk scores such as SCORE2-OP and QRISK3 are used in cardiovascular medicine to predict 10-year event risk and guide preventive interventions (4, 5). In oncology, models help identify high-risk populations (6), aid in early diagnosis (7), predict pathological complete response (pCR) to treatments like neoadjuvant chemoradiotherapy (8), and assess patient recurrence risk and overall survival (3, 9). These applications promise to enhance clinical efficiency and tailor treatments, ultimately improving patient outcomes.

However, the path from model development to clinical application is fraught with challenges, centered not only on discrimination but also on model robustness, calibration, generalizability, and clinical utility. A model that performs exceptionally on its development dataset may experience a sharp performance decline when applied to new, independent populations—a phenomenon termed “illusory generalizability” (10). This problem is evident in oncology. For example, external validation of 87 breast cancer clinical prediction models showed marked variation in performance when these models were applied to independent registry data (11). Similarly, the PREDICT breast cancer prognostic model showed miscalibration and limited clinical utility in young, node-negative women who had not received systemic treatment (12). In ovarian cancer diagnosis, external validation of models for adnexal masses helped identify which models provided more reliable discrimination and calibration across clinical settings (13). In colorectal cancer risk prediction, several models showed acceptable discrimination, but absolute risk estimates required country-specific recalibration before they could be meaningfully reported for individual patients (14). These examples show that external validation, calibration, and clinical utility can substantially change how a model should be interpreted. Such findings highlight the “gatekeeper” role of statistics in guiding models from research to the bedside.

**Figure 1:**
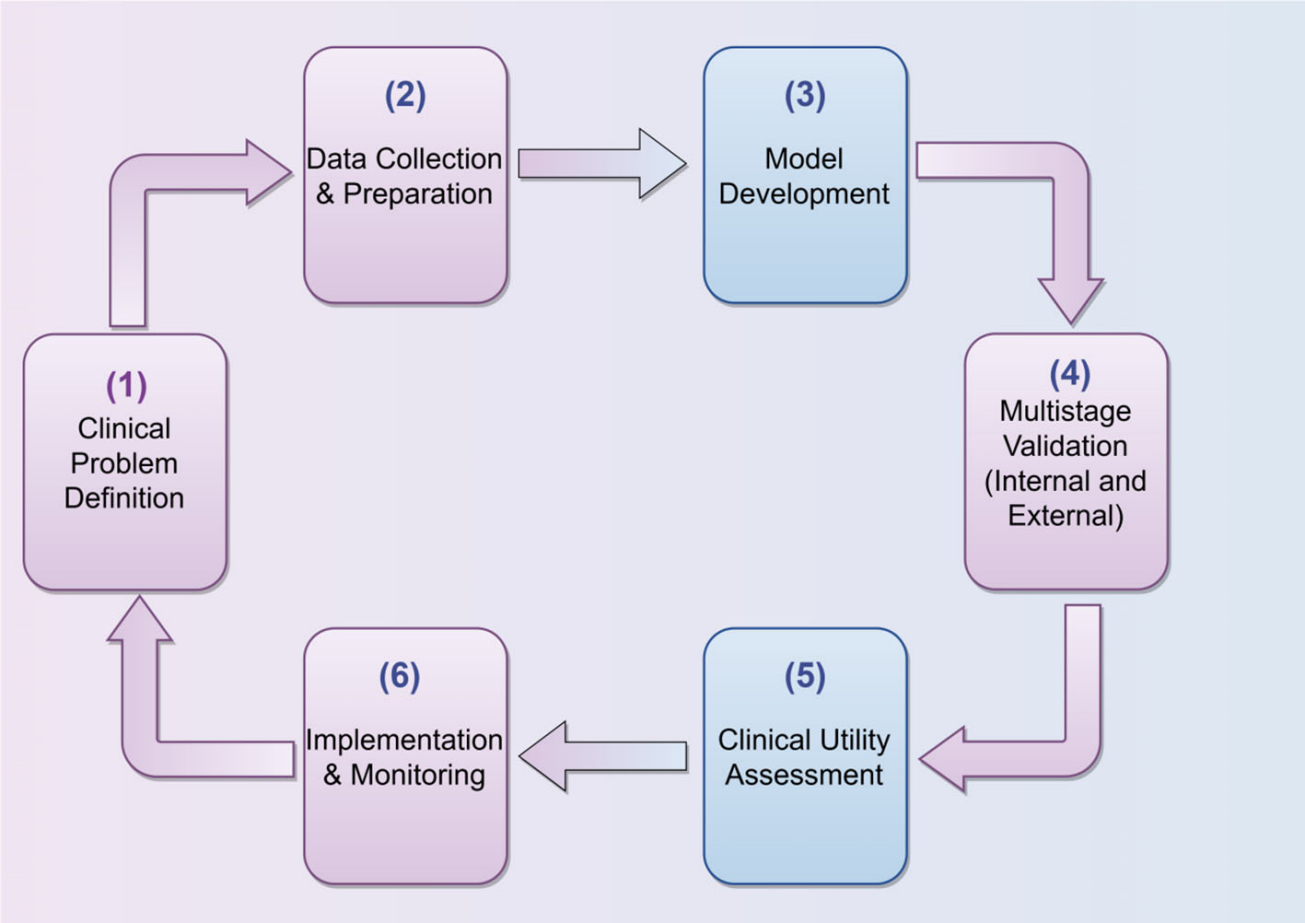
The prediction model lifecycle. A flowchart illustrating the key stages of developing, validating, and implementing a clinical prediction model. The process begins with (1) Clinical Problem Definition, proceeds through (2) Data Collection and Preparation, (3) Model Development, (4) Multi-stage Validation (Internal and External), (5) Clinical Utility Assessment, and concludes with (6) Implementation and Monitoring.

Statistical rigor serves as both the foundation for building prediction models and the primary safeguard of their scientific validity. Every stage, from data preprocessing and feature selection to validation and reporting, depends on sound statistical methods to control bias, assess uncertainty, and prevent overfitting. This framework is also vital for distinguishing prediction (what will happen) from causal inference (why it happens)—a critical distinction to avoid flawed clinical interpretations. Without stringent statistical validation, a prediction model can yield misleading conclusions, lead to flawed clinical decisions, and ultimately harm patients. This entire process, from problem definition to clinical implementation, can be visualized as a lifecycle (**Figure 1**). Therefore, this review aims to fill a critical gap by providing a prescriptive guide that moves beyond a simple description of methods. We offer a comparative framework for algorithm selection in different oncological contexts, a focused discussion of common methodological challenges, and concrete examples for applying reporting standards such as the original TRIPOD statement and its updated TRIPOD+AI guidance (15, 16). To address the need for more actionable guidance, we also introduce a scenario-specific decision framework for selecting appropriate modeling, validation, calibration, and implementation strategies across common oncology prediction settings. As a narrative review, this article synthesizes methodological literature and representative oncology examples rather than conducting a formal systematic review or meta-analysis.

## 2 Building the prediction model: a unified workflow

Building a prediction model is a systematic process that transforms raw data into a clinical tool. Statistics underpins this entire workflow, ensuring the scientific rigor of each step. The performance of a prediction model is highly dependent on the quality of its input data. Data preprocessing is the first step, aiming to clean, transform, and normalize raw data to establish a solid foundation for subsequent analysis. This step is particularly important when dealing with high-dimensional data, particularly image-derived and radiomics data, where overfitting and feature instability are common challenges (17). In medical imaging, image segmentation is a key preprocessing step that precisely delineates the region of interest (ROI), such as a tumor. Deep learning models such as nnU-Net have become robust tools for biomedical image segmentation and have been applied to automate tumor segmentation in pediatric Wilms’ tumor CT (18, 19).

Feature selection is a core task following preprocessing. Its goal is to screen a subset of the most predictive variables from thousands of candidates to simplify the model, reduce computational complexity, prevent overfitting, and generate hypotheses about underlying biological mechanisms (20). Common methods include filter methods, which operate independently of model training and screen variables based on statistical properties such as variance, correlation, chi-squared tests, or mutual information; wrapper methods, which select feature subsets by evaluating model performance across different variable combinations; and embedded methods, which incorporate feature selection within the model training process.

The Least Absolute Shrinkage and Selection Operator (LASSO) regression is one of the most widely used embedded methods (21). By applying an L1-norm penalty to coefficients, LASSO can shrink the coefficients of less informative variables to zero, thus performing feature selection and model construction simultaneously. LASSO has been used to select clinical and radiomic predictors in models for predicting pathological complete response (pCR) in locally advanced rectal cancer (22). However, its use in high-dimensional oncology data requires careful qualification. In omics or radiomics settings, the number of predictors may greatly exceed the number of patients, and predictors are often highly correlated. Under these conditions, LASSO-selected features may be unstable and should not be interpreted as reproducible biomarkers without further assessment. Elastic Net, which combines LASSO and Ridge penalties, may be preferable when correlated predictors are expected (23). Recent omics and radiomics studies further emphasize that feature stability and reproducibility are essential before selected variables are used for biological interpretation or clinical translation (24, 25).

Advanced machine learning methods are also used for feature selection and model development, especially in precision oncology, where multimodal data require efficient processing (2, 20). For example, Random Forest can screen variables by calculating feature-importance scores, while gradient boosting methods can capture non-linear relationships and interactions. However, these models require careful tuning, adequate sample size, and rigorous validation. A systematic review of machine-learning prognostic models in oncology showed that inadequate validation and high risk of bias remain common barriers to clinical translation (26). **Table 1** provides a comparative framework to guide algorithm selection, contrasting common methods based on interpretability, data requirements, ability to model non-linearity, and suitability for specific oncological data types.

**Table 1:** Comparison of common prediction algorithms.

Algorithm	Interpretability	Data requirements (sample size, dimensionality)	Handles non-linearity and interactions	Key strength	Use when… (oncology examples)
Logistic Regression	High (coefficients are directly interpretable as log-odds)	Moderate sample size needed; sensitive to high dimensionality (p >> n)	No (requires manual specification of interaction or polynomial terms)	Simplicity, transparency, and easy clinical implementation	Developing a diagnostic or risk classification model with a small set of pre-specified clinical variables, such as age, stage, laboratory markers, or ECOG score.
Cox Proportional Hazards Model	High (hazard ratios are clinically interpretable)	Requires time-to-event data with adequate follow-up and event numbers	Limited unless interactions or time-varying effects are specified	Foundational method for censored survival outcomes	Predicting overall survival, disease-free survival, progression-free survival, recurrence-free survival, or time to recurrence in oncology.
LASSO/Elastic Net	Moderate (selects a sparse set of predictors, but coefficients may be unstable or biased)	Can handle high dimensionality (p > n), but requires careful validation and stability assessment	No (inherently linear unless non-linear terms are created)	Embedded feature selection for high-dimensional data	Selecting candidate prognostic genes from transcriptomic panels or important features from radiomics data; selected features should not be interpreted as reproducible biomarkers without further validation.
Random Forest (RF)	Low (“black-box”, but feature importance can be ranked)	Can handle mixed and high-dimensional data, but still requires careful tuning and validation on small samples	Yes (captures complex non-linear relationships and interactions)	Robust exploratory model for complex data	Exploring novel biomarkers in omics or radiomics data where complex interactions are expected.
Gradient Boosting (XGBoost, LightGBM)	Low (“black-box”, but SHAP or LIME can provide local explanations)	Often benefits from adequate sample size, careful feature engineering, and hyperparameter tuning; can overfit if not carefully tuned	Yes (highly effective at capturing complex patterns)	Strong predictive performance on tabular data	Building high-performance models integrating clinical, laboratory, imaging, and genomic variables for treatment response or risk prediction.
Convolutional Neural Network (CNN)	Very low (“black-box”, saliency maps or attribution methods may highlight important regions)	Requires large imaging or pathology datasets, or carefully designed transfer learning and data augmentation strategies, plus external validation	Yes (hierarchical feature learning for spatial data)	State-of-the-art method for image analysis	Classifying tumor subtypes from histopathology slides or detecting lesions and metastases in radiological images such as CT or MRI.

The choice between a traditional statistical model and a machine learning model involves a trade-off between interpretability, performance, endpoint type, sample size, and data dimensionality. For a clinical risk score based on a small set of pre-defined predictors, logistic regression is often preferable because it is transparent and easy to implement. In contrast, when exploring high-dimensional data such as genomics or radiomics for novel biomarkers, machine learning methods may help capture complex interactions and rank feature importance, provided that their performance is rigorously validated and their predictions are examined using interpretability tools such as SHAP or LIME.

Other common model types include: (1) Cox Proportional Hazards Model: The Cox proportional hazards model remains the foundational method in survival analysis for predicting time-to-event outcomes, such as death, recurrence, disease progression, or treatment failure (27). It handles censored data and incorporates follow-up time, making it especially important for oncology endpoints such as overall survival, disease-free survival, progression-free survival, and time to recurrence. Many classic cancer prognostic models, such as the Burkitt Lymphoma International Prognostic Index (BL-IPI) (28) and models for postoperative recurrence and survival in renal cell carcinoma (29), are built using Cox regression. Cox-based models should also be assessed by discrimination, calibration, and clinical usefulness, especially during external validation (30). (2) Machine Learning (ML) Models: With increasing data dimensionality and complexity, ML models are commonly used for their powerful pattern-recognition capabilities. These include Support Vector Machines (SVM), Random Forests (RF), and Gradient Boosting Machines (GBM) such as XGBoost and LightGBM. They can capture non-linear relationships and complex interactions, but their apparent performance may be overly optimistic without appropriate validation (26). (3) Deep Learning (DL) Models: Neural network-based models have shown substantial potential in handling images, sequential data, and complex multimodal data (1). Convolutional Neural Networks (CNNs) are central to image analysis (1, 9, 18), while multimodal deep learning, which integrates imaging, pathology, genomics, and clinical variables, is an important trend in oncology prediction research (3, 8, 9). These models may achieve strong performance, but they usually require large datasets, external validation, and interpretability assessment before clinical use.

The model-building process typically involves splitting the dataset into training, validation, and test sets. The training set is used to fit model parameters, the validation set to tune hyperparameters, and the test set for final, unbiased performance evaluation. In oncology studies, this process should be supplemented by resampling-based internal validation and, whenever possible, independent external validation before clinical implementation.

## 3 Evaluating model performance: a multi-faceted framework

A prediction model, regardless of its internal complexity or apparent performance on training data, is of limited clinical value without rigorous validation (30, 31). Model validation is the core process of assessing a model’s performance, including discrimination, calibration, clinical utility, and generalizability (30, 32). The validation framework is primarily divided into internal and external validation.

### 3.1 The challenge of overfitting and the rationale for validation

Overfitting is one of the most common challenges in prediction model research, especially when dealing with high-dimensional data where the number of predictors far exceeds the number of patients (17). An overfitted model learns noise and dataset-specific patterns rather than generalizable clinical signals, leading to excellent performance in the training data but a sharp decline in new patients (10, 30). Statistical strategies to control overfitting include regularization, feature reduction, cross-validation for hyperparameter tuning, bootstrap-based optimism correction, and ensemble learning methods (21, 25, 26, 30, 33).

### 3.2 Internal validation for stability and optimism correction

Internal validation aims to assess model performance within the original development dataset and to correct for performance overestimation caused by overfitting (30, 33). Common internal validation methods include: (1) Cross-validation: The dataset is split into k folds; the model is trained on k-1 folds and tested on the remaining fold. This process is repeated k times, and the final performance is averaged across folds. Five-fold or ten-fold cross-validation is commonly used for model tuning and preliminary stability assessment (30). (2) Bootstrap resampling: This method involves drawing samples with replacement from the original dataset to generate multiple bootstrap samples of the same size. The model is refitted in each bootstrap sample and tested against the original dataset. The difference between bootstrap and original-sample performance is used to estimate optimism and adjust performance metrics, such as AUC or C-index, to obtain a more realistic estimate (33). However, standard bootstrap procedures may still overestimate performance in small samples, a frequent challenge in oncology prediction studies. Bias-corrected bootstrap approaches or the 0.632+ bootstrap estimator may provide more reliable optimism-corrected estimates in such settings (33). Bootstrap resampling can also be used to assess model stability and calculate confidence intervals. Internal validation is a necessary step in model development, but it cannot fully replace external validation for assessing generalizability (30, 32, 34).

### 3.3 External validation as a key test of generalizability

External validation is the most direct test of a model’s generalizability, requiring testing in one or more cohorts that are independent of the development data (32, 34). It should generally precede broad clinical implementation. External validation can be categorized as:

Temporal Validation: Using data collected at the same center but at a later time.

Geographical Validation: Using data from different medical centers or geographical locations. This is a more stringent test of robustness, especially in oncology, where imaging protocols, pathology workflows, treatment standards, and patient case mix may vary across institutions. High-quality oncology studies increasingly emphasize multicenter external validation (7, 8, 11–14, 29).

Transportability Assessment: Evaluating model performance in populations with characteristics that differ from the development cohort, such as ethnicity, tumor subtype, disease stage, treatment era, or healthcare system (10, 30, 32, 34).

A drop in performance during external validation is not necessarily a failure, but an expected and informative outcome. To make this process actionable, researchers should conduct a root-cause analysis by comparing the development and validation cohorts in terms of case mix, measurement processes, treatment patterns, outcome definitions, and missing-data patterns. Stratifying performance by key subgroups can help identify where the model is failing. Such analyses help define the model’s specific context of use and indicate whether recalibration, model updating, retraining, or restriction to a narrower target population is needed (34, 35). Transparently reporting performance degradation is therefore essential for understanding a model’s true generalizability.

### 3.4 A triad of performance metrics: discrimination, calibration, and clinical utility

Evaluating a model requires a comprehensive assessment beyond a single metric. The three key facets of performance are discrimination, calibration, and clinical utility (30, 36–38). Internal validation should first estimate optimism-corrected discrimination, such as AUC or C-index. External validation should then assess whether discrimination and calibration are preserved in independent data. Finally, before clinical implementation, Decision Curve Analysis (DCA), or another formal clinical-utility assessment, should be used to determine whether the model provides a net benefit over existing strategies (37, 38). A model with high AUC but poor calibration and no net benefit should not be considered ready for clinical use without further refinement, recalibration, or updating (35, 36, 38).

**Figure 2:**
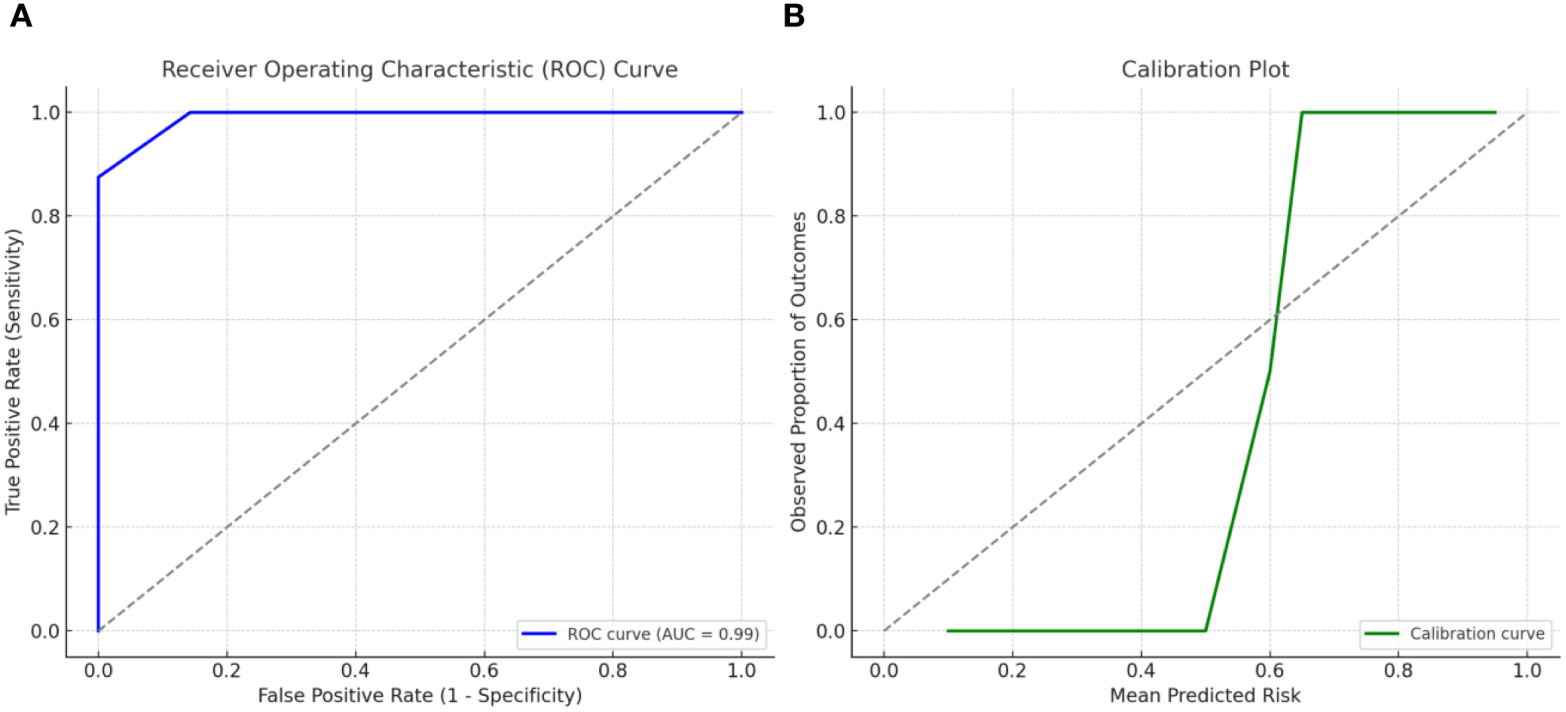
Assessing model performance: discrimination vs. calibration. **(A)** A sample Receiver Operating Characteristic (ROC) curve plots the true positive rate (sensitivity) against the false positive rate (1-specificity) at various classification thresholds. The Area Under the Curve (AUC) quantifies the model’s overall ability to discriminate between patients with and without the outcome. **(B)** A corresponding calibration plot for the same model. To create this plot, patients are typically grouped by deciles of predicted risk. The mean predicted risk for each group (x-axis) is plotted against the observed proportion of outcomes in that group (y-axis). The plot shows how well the model’s predictions align with reality. The calibration slope and intercept quantify systematic errors in prediction. A model with high AUC can still be poorly calibrated, leading to inaccurate risk estimates.

Discrimination refers to the model’s ability to differentiate between individuals with different outcomes. For binary classification, the Area Under the Curve (AUC) summarizes the Receiver Operating Characteristic (ROC) curve. An AUC value of 0.5 indicates no discriminatory ability, whereas a value of 1.0 indicates perfect discrimination (2, 8, 37). The ROC curve visually illustrates how sensitivity and specificity trade off across classification thresholds (**Figure 2A**). For survival outcomes, the Concordance Index (C-index) extends the concept of AUC by assessing agreement between predicted risk rankings and observed event times (29, 37, 39).

Sensitivity refers to the model’s ability to identify true positive cases, whereas specificity refers to its ability to identify true negative cases. These metrics are especially important in diagnostic contexts (6, 37). Calibration evaluates the agreement between predicted probabilities and observed event frequencies. A model may show high discrimination but poor calibration, leading to systematic overestimation or underestimation of risk (36, 37). Calibration plots compare mean predicted risk with observed event frequency across patient subgroups; in an ideally calibrated model, the points align with the 45-degree diagonal line (36). Comparing a calibration plot with a ROC curve for the same model shows that strong discrimination does not necessarily imply adequate calibration (**Figure 2B**).

**Figure 3:**
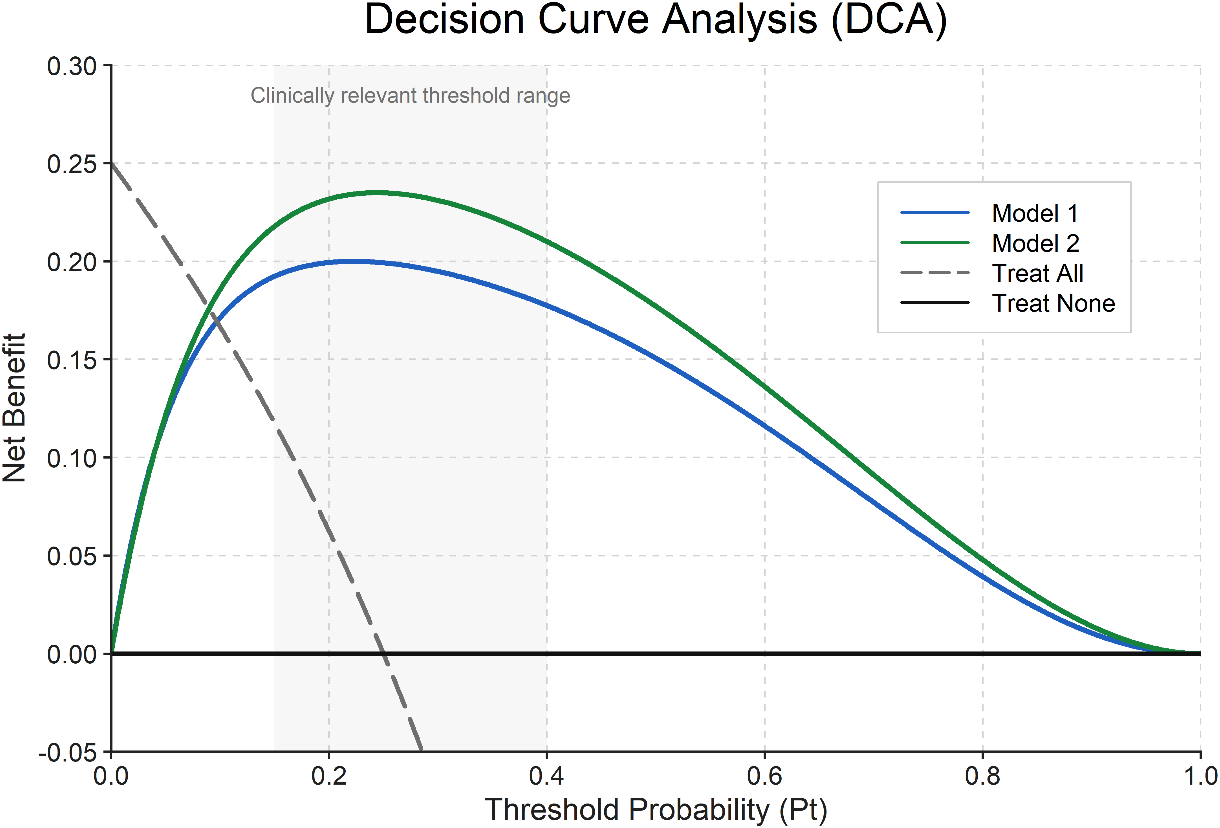
Interpreting decision curve analysis (DCA). This schematic DCA plot compares the net benefit of two prediction models with the default strategies of “treat all” and “treat none”. Net benefit is calculated as (True Positives/N) - (False Positives/N) × (Pt/(1-Pt)), where Pt is the threshold probability and N is the total sample size. The “treat none” strategy is shown as a horizontal line at net benefit = 0, whereas the “treat all” strategy varies with the threshold probability according to the event rate and threshold weighting. The shaded area indicates an illustrative clinically relevant threshold range. A model is considered clinically useful if its decision curve lies above both default strategies across this range.

Clinical utility evaluates whether using the model in clinical practice provides benefit compared with current strategies. DCA quantifies the net benefit of using a model to guide interventions across clinically relevant risk thresholds and compares it with default strategies such as “treat all” or “treat none” (38). A model is considered clinically useful only if its decision curve provides higher net benefit than these default strategies within the threshold range relevant to clinical decision-making. A schematic DCA plot is provided to compare models and illustrate which model offers superior clinical benefit across clinically relevant threshold probabilities (**Figure 3**).

## 4 From research to practice: reporting, advanced topics, and special scenarios

To enhance the transparency and reproducibility of prediction model studies, reporting should adhere to internationally recognized guidelines. Building on the original Transparent Reporting of a multivariable prediction model for Individual Prognosis Or Diagnosis (TRIPOD) statement, the updated TRIPOD+AI guidance provides current reporting recommendations for prediction model studies using regression or machine-learning methods (15, 16). A systematic review of machine-learning prognostic models in oncology showed that incomplete reporting and methodological weaknesses remain common, particularly in areas such as sample size justification, missing data handling, validation, and performance reporting (26). The PROBAST tool is used to assess the risk of bias and applicability of prediction model studies (31). Reviews show that many published AI and machine learning models have a high risk of bias, primarily in the analysis domain, such as insufficient sample size and lack of correction for overfitting (26, 31).

To further aid researchers, we summarize common methodological pitfalls and their corresponding solutions in **Table 2**. These pitfalls, ranging from flawed validation to inadequate reporting, represent major barriers to clinical translation. A critical issue, especially for complex “black-box” models, is the lack of interpretability reporting. Tools such as SHAP and LIME can help explain individual predictions and identify influential variables (40, 41). However, such explanations should not be interpreted as causal evidence and cannot replace external validation, calibration assessment, or evaluation of clinical utility. In practice, the choice between a high-performing but opaque model and a simpler interpretable model should depend on the clinical context; in high-stakes decisions, a modest gain in discrimination should not outweigh the need for robust external validation, good calibration, clinical net benefit, and clinically plausible explanations. A concise checklist for reporting and appraising oncology prediction models is provided in **Box 1**.

**Table 2:** Common pitfalls in prediction model research and recommended solutions.

Pitfall	Description	Recommended Solution
1. Overly Optimistic Performance	Reporting performance on the training set or relying on simple data splits, leading to inflated metrics due to overfitting.	Implement rigorous internal validation, such as k-fold cross-validation, nested cross-validation for model tuning when feasible, and bootstrap resampling, and report optimism-corrected performance where appropriate.
2. Fixation on Discrimination	Relying solely on AUC or C-index to judge a model, while ignoring whether predicted risks are accurate in magnitude.	Report the performance triad: discrimination, such as AUC or C-index with 95% CI; calibration, such as calibration plot with slope/intercept; and clinical utility, such as Decision Curve Analysis.
3. Neglecting External Validation	Assuming that a model with good internal performance will generalize to new populations, which can lead to illusory generalizability.	Conduct external validation in at least one independent cohort, preferably from a different time period or medical center. Transparently report performance changes and analyze possible causes, such as case mix, measurement processes, treatment patterns, outcome definitions, and missing-data patterns.
4. Naive Handling of Missing Data	Using complete-case analysis without justification, which can reduce sample size and introduce bias.	Use principled imputation methods, such as Multiple Imputation by Chained Equations (MICE), when appropriate, and clearly report the missing-data strategy according to TRIPOD/TRIPOD+AI recommendations (15, 16).
5. The Black-Box Problem	Deploying complex models, such as deep learning or gradient boosting, without understanding how predictions are generated, thereby limiting clinical trust and adoption.	Use interpretability tools such as SHAP or LIME to explain individual predictions and summarize influential predictors at the model level when appropriate. These explanations should support, not replace, external validation, calibration assessment, and clinical utility evaluation.
6. Irreproducible Research	Publishing results without enough detail for others to replicate the analysis, validate the model, or apply it to new patients.	Follow reporting guidelines such as TRIPOD and TRIPOD+AI (15, 16). Provide the full model when applicable, such as regression coefficients, and share analysis code, preprocessing details, and trained model information when possible.

Box 1 **Best practices and reporting checklist for oncology prediction models informed by TRIPOD, TRIPOD+AI, and PROBAST.**

A concise checklist summarizing key recommendations for researchers, with concrete reporting examples for a hypothetical model predicting 5-year survival in colorectal cancer (CRC).

**1. Comprehensive validation is mandatory.**

Internal Validation: Report optimism-corrected performance, such as bootstrap-corrected estimates, to mitigate overestimation caused by overfitting.

External Validation: Test the model in at least one independent cohort. Geographical validation using data from different centers is preferred when available, while temporal validation using later data from the same center can also provide useful evidence. Transparently report any performance changes, such as shifts in C-index or AUC, and analyze possible drivers, including differences in case mix, imaging protocols, treatment patterns, outcome definitions, and missing-data patterns.

**2. Report the performance triad.**

Discrimination: For survival outcomes, such as CRC 5-year survival, report C-index with 95% confidence intervals (CIs). For binary outcomes, such as pathological complete response, report AUC with 95% CIs. Include metrics for development, internal validation, and external validation datasets when applicable.

Calibration: Present a calibration plot and report calibration slope and intercept when possible to quantify the agreement between predicted risks and observed event frequencies.

Clinical Utility: Include Decision Curve Analysis (DCA) and justify clinically relevant risk thresholds, such as thresholds based on balancing overtreatment harm against undertreatment risk.

**3. Adhere to TRIPOD/TRIPOD+AI and assess risk of bias.**

Use TRIPOD and TRIPOD+AI to guide transparent reporting of prediction model studies and use PROBAST to assess risk of bias and applicability.

Hypothetical example – Missing data reporting: “Of 850 patients in the development cohort, serum carcinoembryonic antigen (CEA) was missing for 62 (7.3%). We used multiple imputation with chained equations (MICE) under the missing-at-random assumption, creating 20 imputed datasets. The imputation model included age, sex, tumor TNM stage, and 5-year survival outcome”.

Hypothetical example – Model performance reporting: “In the external validation cohort (n=410, multi-center CRC patients), the model had a C-index of 0.78 (95% CI: 0.74–0.82) for discrimination. The calibration plot showed slight high-risk overestimation (calibration slope = 0.91). DCA demonstrated net benefit over ‘treat all’ for risk thresholds 15%–60%”.

**4. Ensure reproducibility and interpretability.**

Model/Code Access: For regression models, such as Cox models, provide full equations with coefficients or hazard ratios. For machine learning models, provide preprocessing details, hyperparameters, validation workflow, analysis code, or trained model information when possible.

Interpretability: Use tools such as SHAP or LIME to explain “black-box” predictions and report feature importance when appropriate. In high-stakes clinical settings, a modest gain in discrimination may not justify loss of transparency unless the model also demonstrates robust external validation, good calibration, clinical net benefit, and clinically plausible explanations.

Reproducibility is a cornerstone of trustworthy prediction model research. To allow others to validate or use a model, reports should provide sufficient detail, including patient characteristics, eligibility criteria, preprocessing steps, predictor definitions, model specification, and analysis code when possible. When patient-level data cannot be shared because of privacy restrictions, authors should still provide enough methodological detail, model parameters, or validation code to support independent appraisal.

Advanced topics are also shaping the field. Federated learning offers a potential solution to data privacy challenges in multicenter studies by allowing model training across institutions without sharing raw patient data (42). While promising, it does not eliminate statistical heterogeneity across centers, such as differences in patient populations, imaging protocols, pathology workflows, treatment standards, or outcome definitions. Site-specific recalibration, federated transfer learning, or meta-analytic combination of site-level models may be needed.

### 4.1 Methodological considerations for special scenarios in oncology

Modern oncology prediction faces several unique challenges that require tailored methodological approaches. Rare Tumors: Model development for rare cancers is limited by small sample sizes, which increases the risk of overfitting and unstable estimates. Possible strategies include Bayesian methods, transfer learning from larger related datasets, and data sharing through national or international consortia. In this setting, researchers should report uncertainty, avoid unnecessary model complexity, and prioritize cross-center validation.

Real-World Data (RWD) Models: Models built using electronic health records or registries may improve generalizability but are susceptible to specific biases. Immortal time bias can occur when time between cohort entry and treatment initiation is incorrectly attributed to the exposed group. Selection bias may arise when the study population is not representative of the target population, and missing data are common. For RWD-based oncology models, researchers should clearly define the time origin, eligibility criteria, exposure windows, outcome definitions, and missing-data strategy.

Dynamic Prediction Models: Many oncology decisions require predictions that evolve as new information becomes available, such as treatment response, biomarker changes, toxicity, or radiologic progression. Static models provide a single risk estimate at baseline, whereas dynamic models update risk over time. Landmarking and joint modeling are two common approaches. Because these models provide time-updated predictions, validation should also be dynamic, using metrics such as time-dependent AUC, time-dependent calibration, and clinically relevant prediction horizons.

To translate these methodological principles into practical decisions, **Table 3** summarizes scenario-specific modeling, validation, and reporting considerations for common oncology prediction tasks. This framework is intended as a practical guide rather than a rigid hierarchy; the final modeling strategy should be determined by the clinical question, endpoint type, sample size, data dimensionality, validation setting, and intended clinical use.

**Table 3:** Scenario-specific decision framework for oncology prediction models.

Oncology prediction scenario	Preferred modeling strategy	Validation and performance priorities	Main caution and recommended action
Binary clinical outcomes with limited predictors, such as cancer diagnosis, treatment toxicity, pCR, or high-risk classification	Logistic regression or penalized logistic regression when the number of predictors is large relative to events	Internal validation using cross-validation or bootstrap; external validation when possible; report AUC, calibration, and DCA (30, 33, 34, 37, 38)	Avoid excessive predictors and purely data-driven variable selection. Prioritize transparent coefficients, calibration, and clinically meaningful thresholds.
Time-to-event outcomes, such as OS, DFS, PFS, recurrence-free survival, or time to recurrence	Cox proportional hazards model; consider penalized Cox models when predictors are numerous	Assess C-index, time-specific calibration, censoring structure, and external validation; check whether model performance changes across centers or treatment eras (27, 29, 36, 37, 39)	Do not treat survival outcomes as simple binary endpoints without justification. Consider proportional hazards assumptions and clinically relevant prediction horizons.
High-dimensional radiomics or omics prediction	LASSO, Elastic Net, or carefully tuned machine learning models	Use internal validation, feature-stability assessment using bootstrap or repeated resampling, calibration, and independent external validation (22–26, 30, 34)	Selected features may be unstable, especially with small samples and correlated predictors. Avoid interpreting selected variables as reproducible biomarkers without stability assessment and external validation.
Image-based or pathology-based prediction	CNNs or other deep learning models; combine with clinical variables when appropriate	Multicenter external validation across scanners, protocols, and institutions; report discrimination, calibration, and interpretability analyses (1, 9, 18, 19, 34, 40, 41)	Strong internal performance may reflect scanner, staining, or site-specific artifacts. Use explainability tools cautiously, assess possible data leakage, and consider preprocessing standardization or harmonization across sites.
Multimodal prediction integrating clinical, imaging, pathology, and molecular data	Machine learning, deep learning, or hybrid models with transparent reporting of data integration	Validate each modality-specific preprocessing step; report missing-data handling, calibration, clinical utility, and external performance (3, 8, 9, 30, 31)	Complex multimodal models may overfit and may not generalize if one modality is unavailable or measured differently in external cohorts.
Rare tumors or very small datasets	Simple models, penalized regression, Bayesian methods, transfer learning, or consortium-based pooled analyses	Emphasize uncertainty, bootstrap validation, cross-center validation, and calibration rather than only discrimination (30, 33, 34)	Avoid overly complex models. Report uncertainty clearly and prioritize collaborative data sharing or external validation through rare-tumor consortia.
Real-world data models using EHRs, registries, or claims data	Transparent models or machine learning models with explicit bias control	Define time origin, exposure windows, outcome definitions, and missing-data strategy; assess temporal and external validation, calibration, and sensitivity analyses (30, 32, 34)	Watch for immortal time bias, selection bias, measurement error, and informative missingness. Do not rely on complete-case analysis without justification.
Dynamic prediction during follow-up or treatment	Landmarking, joint modeling, or time-updated machine learning models	Use time-dependent AUC, time-dependent calibration, and clinically relevant prediction horizons (36, 37, 39)	Static baseline validation is insufficient. The model should be evaluated at the time points where predictions will actually inform clinical decisions.
Privacy-constrained multicenter modeling	Federated learning, federated transfer learning, or site-level model aggregation	Evaluate site-specific and pooled performance; assess calibration across centers and consider site-specific recalibration (42)	Federated learning protects data privacy but does not eliminate heterogeneity. Differences in case mix, imaging protocols, and treatment standards still require explicit validation.
Models intended for clinical implementation	Prefer models with clear clinical use cases, predefined decision thresholds, and implementation plans	Evaluate clinical utility using DCA and assess whether model use improves decisions across clinically relevant thresholds (38)	A statistically accurate model may still be clinically unhelpful. Confirm threshold-specific net benefit, workflow feasibility, user-facing implementation, safety, and post-deployment performance monitoring before implementation.

This framework synthesizes methodological recommendations discussed in the preceding sections, including guidance on model development, external validation, performance evaluation, model updating, reporting, and clinical utility (30, 31, 34–38). It is intended as a practical guide rather than a rigid hierarchy. The final modeling strategy should be determined by the clinical question, endpoint type, sample size, data dimensionality, validation setting, and intended clinical use. pCR, pathological complete response; OS, overall survival; DFS, disease-free survival; PFS, progression-free survival; EHRs, electronic health records; AUC, area under the curve; DCA, decision curve analysis; CNN, convolutional neural network.

## 5 Discussion and future outlook

Prediction models hold great promise in oncology and other medical fields, bridging large datasets and individualized clinical decisions. However, model development is only the first step; scientific and clinical value is ultimately determined by rigorous validation. This review has outlined a statistical framework for this process, emphasizing the distinct but complementary roles of internal and external validation. While internal validation is key for controlling overfitting and obtaining initial performance estimates, external validation—particularly in multicenter and diverse populations—is a critical test of a model’s generalizability and robustness (30, 34).

A critical takeaway is the need to move beyond reporting a single discrimination metric. A multifaceted evaluation that includes calibration and clinical utility, such as Decision Curve Analysis, is essential for determining a model’s true value (36–38). It is also crucial to recognize that a drop in performance during external validation is not simply a failure, but an expected and informative outcome. Such degradation may reflect differences in case mix, measurement processes, treatment era, outcome definitions, or missing-data patterns, and can help define the model’s context of use and the need for recalibration, updating, retraining, or more restricted application (34, 35).

Adherence to reporting guidelines is fundamental for ensuring transparency and reproducibility. The original TRIPOD statement and its updated TRIPOD+AI guidance provide structured reporting guidance for prediction model studies, while PROBAST supports assessment of risk of bias and applicability (15, 16, 31). This review has moved beyond a descriptive overview to provide a more prescriptive framework. By introducing a comparative guide for algorithm selection (**Table 1**), summarizing common methodological pitfalls and solutions (**Table 2**), and adding a scenario-specific decision framework for oncology prediction tasks (**Table 3**), we provide a practical roadmap for model development, validation, reporting, and clinical interpretation.

The field faces ongoing challenges, including the high risk of overfitting with high-dimensional data, the limitations of small sample sizes, sources of measurement and selection bias, and the interpretability of complex “black-box” models. Tools such as SHAP and LIME can improve transparency by identifying influential variables or unexpected model behavior (40, 41). However, these explanations should not be interpreted as causal evidence, and they cannot replace external validation, calibration assessment, or evaluation of clinical utility. Future research must be methodologically more rigorous, emphasizing adequate sample size estimation, multicenter collaboration, reproducible workflows, transparent reporting, and privacy-preserving technologies such as federated learning (42).

Building on this foundation of robust predictive modeling, the field is poised to advance toward a more profound, yet distinct, goal: causal inference. While prediction models excel at forecasting outcomes, they do not, by themselves, explain the causal effects of interventions. Misinterpreting a predictive feature as a causal target can lead to ineffective or even harmful clinical strategies. Causal machine learning aims to bridge this gap by estimating individualized treatment effects and supporting personalized treatment decisions (43). Mendelian randomization can provide complementary causal evidence by using genetic variants as instrumental variables; in oncology, recent studies have applied this approach to investigate inherited susceptibility and potentially modifiable risk factors for early-onset colorectal cancer (44). This shift from predicting “what will happen” to exploring “why it happens” may provide deeper insights and guide the development of more targeted interventions.

As a narrative review, this article used a targeted rather than exhaustive literature search, and no formal risk-of-bias assessment or quantitative evidence synthesis was performed. In conclusion, statistics provides the essential foundation and tools for building and validating prediction models. Trustworthy prediction models are built upon the integration of advanced computational methods with rigorous statistical principles. Translating these models into clinical practice depends on comprehensive validation, transparent reporting, scenario-appropriate modeling decisions, and critical assessment of real-world utility. This approach is essential for advancing precision oncology and ensuring that prediction models are not only accurate, but also reliable, interpretable, and clinically useful.
